# Otorhinolaryngology without borders

**DOI:** 10.5935/1808-8694.20130121

**Published:** 2015-10-08

**Authors:** Agrício Nubiato Crespo, Alexandre Caixeta Guimarães

**Affiliations:** Senior Associate Professor; Head of ENT - Medical School of UNICAMP; Otorhinolaryngologist; Graduate student in medical sciences - UNICAMP

We ended 2013 with a historic achievement in Otorhinolaryngology. The flagship journal of the national scientific community has received an impact factor, a unique achievement in Latin America.

The Brazilian Journal of Otorhinolaryngology (BJORL) has been in an upbeat path in the last decade. Having overcome the steps of successive indexations our challenge now reaches international standards. Our journal ceases to be a leading national publication to join the great journals of the world. To maintain and increase its impact factor, it will be necessary to attract relevant papers that generate citations in other publications. We will have to attract authors from other countries, as do most internationally known journals.

## Science Mobility Program

Like the federal government, which implemented a program of sending about 100 thousand students abroad to encourage scientific production in Brazil^1^ the BJORL must find opportunities to attract publications from other countries.

This is no simple task, considering the large number of existing journals and the tradition many of them have earned.

Over the next 12 months, the BJORL will be regularly sent to 100 scientists and opinion leaders around the world, pursuing a higher international visibility strategy.

In order to evaluate our potential, we perform an extensive comparative study between the BJORL traits and those from the leading international journals. Laryngology and voice was our field of choice.

Between 2009 and 2013, we assessed the relevant publications in the BJORL and in the five most traditional journals in this field: Laryngoscope, Otolaryngology-Head and Neck Surgery, JAMA Archives of Otolaryngology-Head Neck Surgery, Journal of Voice and the European Archives of Otorhinolaryngology. These journals were selected by impact factor - obtained from the ranking list of impact factor provided by the journals themselves^2^ - and for being traditional models and established destinations of laryngology publications.

We read 1,487 abstracts published between January 2009 and November 2013, from the Laryngology sections of the Journals or those with laryngology-related titles from the journals that did not have such section.

The abstracts were classified according to their main content within one of the following themes: Vocal Assessment, stem cells, wound healing, dysphagia, dystonia, benign disease, stenosis, diagnostic tests, growth factors, pathophysiology, phonomicrosurgery, cancer, laryngeal papillomatosis, vocal fold paralysis and pharyngolaryngeal reflux.

**Figure 1:**
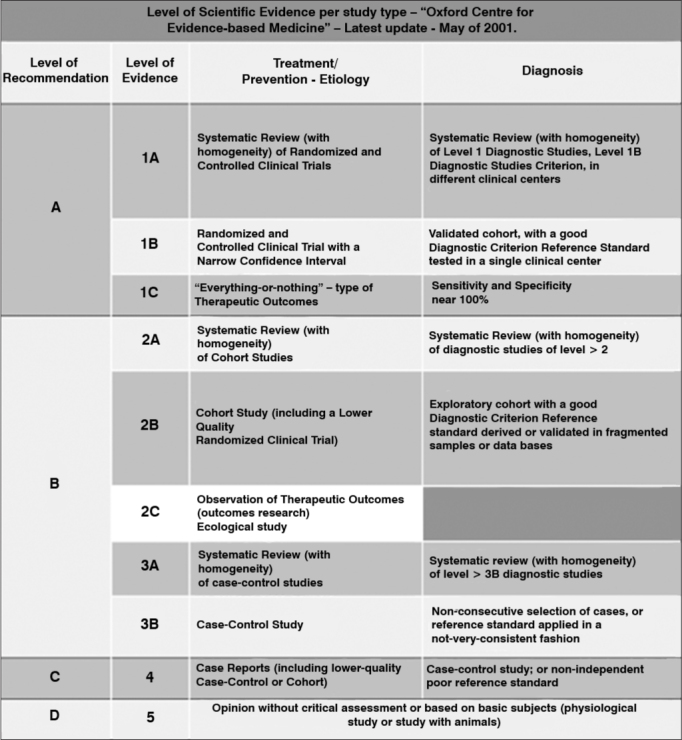
Oxford Centre Classification for the degree of recommendation and level of evidence.

The scientific production of the last five years was evaluated as to the type of study, level of evidence and country of origin, as well as its distribution by subject. The scientific evidence level of each paper was classified according to the Oxford Centre for Evidence-Based Medicine^3^. In their classification, the studies are classified in grade A for those with evidence levels 1A, 1B and 1C; recommendation grade B for those with evidence levels 2A, 2B, 2C, 3A and 3B; grade C recommendation for those with evidence level 4; and grade D recommendation for those with evidence level 5 (Figure 1).

**Figure 2:**
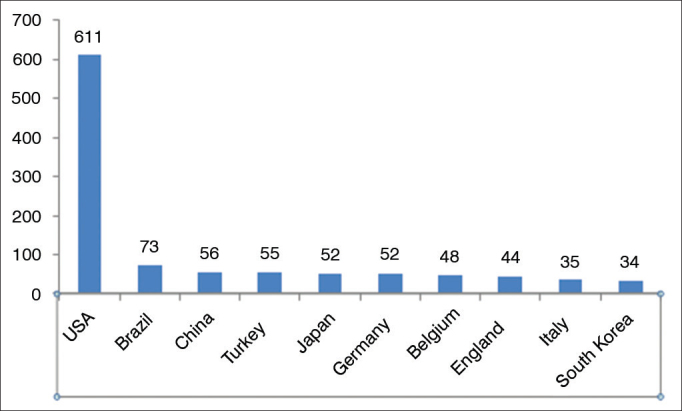
Distribution of the number of papers in laryngology by country in the last five years from the five international journals (total N = 1,487).

The results revealed that Brazil is the second country in the generation of scientific papers in Laryngology and Voice, published in five selected newspapers, second only to the United States, accounting for 41% of the papers published during the assessment period. Brazil is ahead of European and Asian nations such as Germany, Japan, England, France and other traditional strongholds of scientific production (Figure 2).

**Figure 3:**
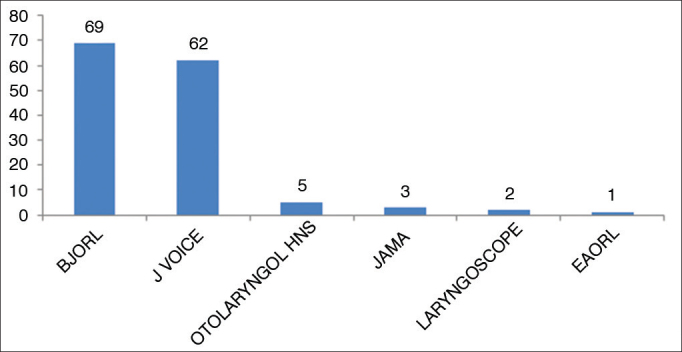
Distribution of Brazilian papers in laryngology 2009–2013.

From this statement we have excluded the papers published by the BJORL, considering that many countries also publish their own journals, where they publish much of their scientific production. Brazilian publications, besides the BJORL, are concentrated in the Journal of Voice, possibly due to its specificity in the subjects of the matter (Figure 3).

**Figure 4:**
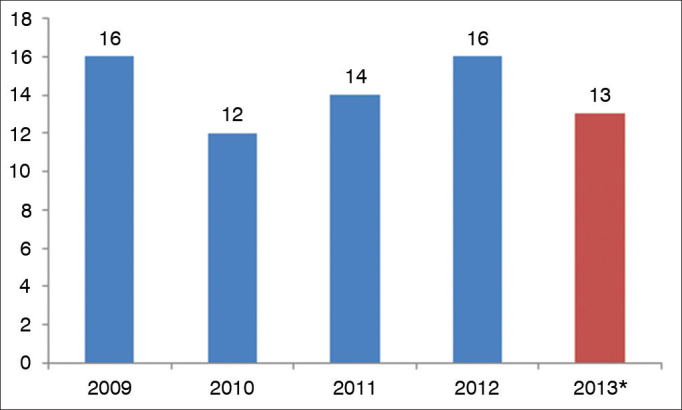
Distribution of the number of publications per year from 2009 to 2013 in the BJORL. ^*^Here we have not included the publications from the last issue of 2013 (November and December).

The number of laryngology publications did not vary much along the years in the study, showing a stable BJORL interest in the area (Figure 4).

**Figure 5:**
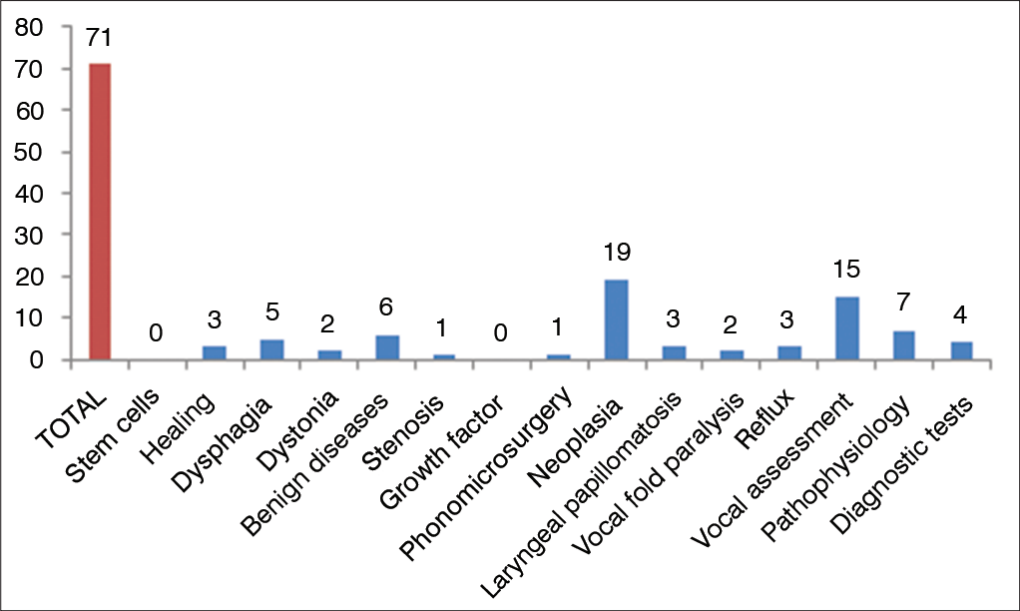
Distribution of BJORL publications by topic in laryngology 2009–2013.

The most publish subjects in the BJORL were neoplasia and vocal assessment. There were no publications on stem cells or growth factor in the study period (Figure 5).

**Figure 6:**
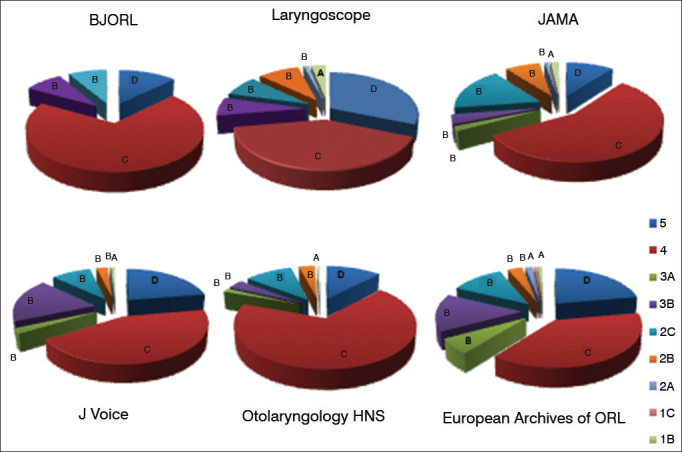
Distribution of publications according to the level of evidence and recommendation grade among the journals between 2009 and 2013.

The BJORL focuses on level-C-recommendation publications. Publications with level B recommendation focus on papers with evidence levels 2C and 3B. The main types of studies were retrospective descriptive; prospective descriptive and case reports. Others, less common types were case-control studies, experimental studies and epidemiological studies on prevalence. In comparison with the other five international journals, there is a higher number of Level-4 publications and no publication with Recommendation Level A in the BJORL (Figure 6).

Although this study was specific to the field of Laryngology, we believe that this information can be useful to guide future directions to our journal.

We conclude that Brazil boasts a significant scientific output in laryngology; we are the second country in the number of publications in the most prestigious international journals. We need to qualitatively improve this large scientific production, producing more studies with evidence Levels A and B.

## Acknowledgments

*Henrique Furlan Pauna Fernando Laffitte Fernandes Carlos Correa*
